# The Reverse Chameleon Effect: Negative Social Consequences of Anatomical Mimicry

**DOI:** 10.3389/fpsyg.2020.01876

**Published:** 2020-07-31

**Authors:** Daniel Casasanto, Laura Staum Casasanto, Tom Gijssels, Peter Hagoort

**Affiliations:** ^1^Department of Human Development, Cornell University, Ithaca, NY, United States; ^2^Department of Psychology, Cornell University, Ithaca, NY, United States; ^3^Department of Psychology, The University of Chicago, Chicago, IL, United States; ^4^Neurobiology of Language Department, Max Planck Institute for Psycholinguistics, Nijmegen, NL, United States; ^5^Donders Institute for Brain, Cognition, and Behavior, Radboud University Nijmegen, Nijmegen, NL, United States

**Keywords:** Chameleon effect, conversation, mimicry, social perception, valence, virtual reality

## Abstract

Bodily mimicry often makes the mimickee have more positive feelings about the mimicker. Yet, little is known about the causes of mimicry’s social effects. When people mimic each other’s bodily movements face to face, they can either adopt a mirrorwise perspective (moving in the same absolute direction) or an anatomical perspective (moving in the same direction relative to their own bodies). Mirrorwise mimicry maximizes visuo-spatial similarity between the mimicker and mimickee, whereas anatomical mimicry maximizes the similarity in the states of their motor systems. To compare the social consequences of visuo-spatial and motoric similarity, we asked participants to converse with an embodied virtual agent (VIRTUO), who mimicked their head movements either mirrorwise, anatomically, or not at all. Compared to participants who were not mimicked, those who were mimicked mirrorwise tended to rate VIRTUO more positively, but those who were mimicked anatomically rated him more *negatively*. During face-to-face conversation, mirrorwise and anatomical mimicry have opposite social consequences. Results suggest that visuo-spatial similarity between mimicker and mimickee, not similarity in motor system activity, gives rise to the positive social effects of bodily mimicry.

## Introduction

People often mimic each others’ bodily movements spontaneously: This tendency to mimic others automatically has been called the *Chameleon Effect* (Chartrand and Bargh, 1999). Being a “chameleon” has social consequences. Typically, mimicking someone causes the mimickee to have more positive feelings about the mimicker (Chartrand and van Baaren, 2009, for review; but see Leander et al., 2012; Liu et al., 2011; Stel et al., 2011).

During face-to-face interactions, people tend to spontaneously mimic other people *mirrorwise*: If the mimickee moves her right hand, the mimicker moves his left hand (Wapner and Cirillo, 1968; Bavelas et al., 1988). However, people can also mimic others *anatomically*: If the mimickee moves her right hand, the mimicker moves his right hand, too. In mirrorwise mimicry, the mimicker’s movements are similar to the mimickee’s visuo-spatially, because they are oriented in the same absolute direction. In anatomical mimicry, the mimicker’s movements are more similar to the mimickee’s motorically: Although their movements may go in different directions, the mimicker and mimickee plan and execute the same actions, using the same effectors, rather than using their homologs on the opposite side of the body. Chartrand and Bargh (1999) posit that creating “similarity” between the mimicker and mimickee is of critical importance for the Chameleon Effect, but it has remained unclear what *kind of similarity* is responsible for the positive consequences of mimicry: visuo-spatial or motoric. Do different kinds of similarity have different social consequences?

To find out, here we contrasted the social consequences of mirrorwise mimicry with those of anatomical mimicry. In order for this comparison to be meaningful, it is essential that the two kinds of mimicry be equated in terms of their precision, naturalness, and timing. To meet this demand, we asked participants to have a face-to-face conversation with a digital human (VIRTUO), in a fully immersive virtual environment (Bailenson and Yee, 2005; Stel et al., 2010). Participants’ spontaneous head movements were tracked using motion capture, and VIRTUO mimicked them after a brief delay, either mirrorwise (in the same absolute direction) or anatomically (in the opposite absolute direction). In a baseline condition, participants were not mimicked and instead saw VIRTUO enact a previous participant’s head movements, which were equally natural but did not bear any systematic relationship to the participants’ own movements. After interacting with VIRTUO, participants answered questions probing how positively they felt about him, as a measure of the social consequences of mimicry.

If the social consequences of mimicry depend on visuo-spatial similarity between the actions of the mimicker and mimickee, then mirrorwise mimicry should have more positive social consequences than anatomical mimicry. Alternatively, if the social consequences of mimicry are due to motoric similarity, then anatomical mimicry should have more positive social consequences than mirrorwise mimicry. Finally, if visuo-spatial and motoric similarity have similar social consequences, then positivity ratings should not differ between the two mimicry conditions: Participants in both the mirrorwise and anatomical mimicry conditions should evaluate VIRTUO more positively than participants in the no-mimicry baseline condition.

## Materials and Methods

### Participants

Native Dutch-speaking participants (*N* = 117, 80 female) from the Radboud University community participated for payment, after giving informed consent. The sample size was not determined a priori; we collected as many participants as possible during the academic year in which the study was run. The experimental program randomly assigned participants to either the Anatomical Mimicry (*N* = 39), Mirrorwise Mimicry (*N* = 40) or No Mimicry (*N* = 38) conditions.

### Apparatus

**FIGURE 1:**
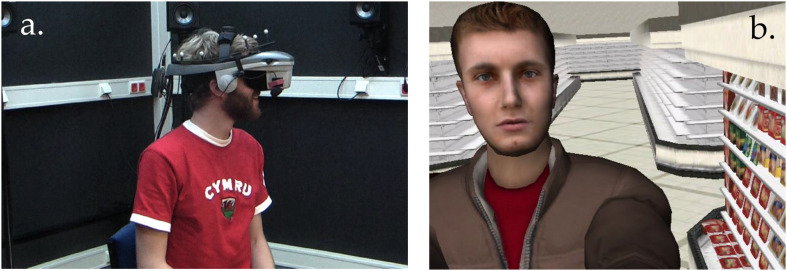
**(a)** Virtual reality set-up used for this experiment. **(b)** Portrait of the virtual agent (VIRTUO) and the virtual supermarket in which the experiment took place.

A virtual environment (VE) was created using Adobe 3ds Max 4^1^ and Vizard software^2^. Participants interacted with a virtual agent named VIRTUO. They wore an NVIS nVisor SX60 head-mounted display (1280 × 1024 resolution, 60′ monocular field of view) outfitted with eight reflective markers linked to a passive infrared DTrack 2 motion tracking system^2^. Sounds in the VE were rendered with a 24-channel WorldViz Ambisonic Auralizer System (Figure 1a). VIRTUO was a WorldViz stock avatar, and appeared to be a Caucasian male in his mid-twenties (Figure 1b). VIRTUO’s speech was pre-recorded by a male native Dutch speaker reading in a conversational tone from a script of statements and questions designed to simulate a conversation.

## Materials and Procedure

Prior to entering the VE, participants were told that they would be having a conversation with VIRTUO, who wanted to learn more about the human world. After the participants were familiarized with the VE, VIRTUO appeared, seated across a table, face to face with the participant, who remained seated throughout the experiment. VIRTUO introduced himself and invited the participants to have a conversation with him about some common items found in human grocery stores (bananas, ketchup, light bulbs, toothpaste, cat food, and beer). He told the participant he was trying to learn more about the human world so that he could act more human. Each item appeared in turn on the table, centered in between the participant and VIRTUO. VIRTUO asked the participants three or four questions about each item (e.g., *My data files suggest this is ketchup, is that right? I’m not programmed with taste buds, but I’d like to try to understand what things taste like. Could you tell me what ketchup tastes like?*). Participants responded with information about the identity of the products, what they were made of, etc.

VIRTUO’s speech created a conversational setting, but he did not have the ability to understand or flexibly respond to participants’ utterances. The experimenter listened to participants’ responses from a control booth, and pressed a button to advance VIRTUO to the next utterance in his script, which began after a delay that varied randomly between 150 and 400 ms. If the next item in VIRTUO’s script did not constitute a sensible response to something a participant said, the experimenter pressed a button that caused VIRTUO to say that he did not understand, and that they should move on.

The experimenter was not aware of the participant’s condition until the experiment had begun, thus experimenter expectancy could not influence participants’ attitudes during their pre-experiment interactions. Participants’ head movements were recorded, and rendered as head movements in VIRTUO at a 2-s delay. Successful mimicry studies have used delays ranging from 1 to 4 s. We chose a 2-s delay (Leander et al., 2012) because, upon piloting different delays on ourselves, the experimenters believed this delay seemed the most natural. In the Anatomical and Mirrorwise mimicry conditions, a participant’s own movements were rendered as VIRTUO’s head movements in real time, causing VIRTUO to mimic the participant’s head movements from either an anatomical or mirrorwise perspective. In the No Mimicry condition, the previous participant’s head movements were rendered. In addition to moving his head, VIRTUO also moved his mouth according to the volume envelope of his (recorded) speech, and blinked his eyes on a random cycle, across all conditions. The conversation lasted 10–15 min.

After exiting the VE, participants completed five questions intended to probe how positively participants felt about VIRTUO following their conversation: “How much did you like VIRTUO?” and “How friendly/funny/attractive/intelligent was VIRTUO?” Participants answered these questions on a scale from 1 (least positive) to 9 (most positive). Responses to these 5 questions were averaged to constitute a Positivity Index, which served as the dependent measure for the experiment. Additionally, participants answered 3 demographic questions and 16 questions that were not relevant for this experiment, but were intended to help us develop the newly created VE for future experiments (e.g., How comfortable was the VR helmet?) Finally, participants completed a debriefing question: Did you notice anything about VIRTUO’s head movements? Some participants commented on the frequency or naturalness of head movements, but no one reported noticing that VIRTUO was mimicking them. The study was approved by the ethics committee of the MPI for Psycholinguistics/Donders Institute.

## Results

Prior to analysis, the data were checked for any violations of the assumptions of linear regression models. No violations were detected. There was no opportunity for multicollinearity, since the there was only one independent variable of interest (Mimicry Condition), which was uncorrelated with the only control variable that was added to the model (the participants’ Gender). There was no visible heteroscedasticity in the distribution of the responses or the residuals, and no deviation from normality according to Shapiro–Wilk tests conducted first on the whole data set and then on each Mimicry Condition separately (all W-values greater than 0.97; all *p*-values greater than 0.44).

**FIGURE 2:**
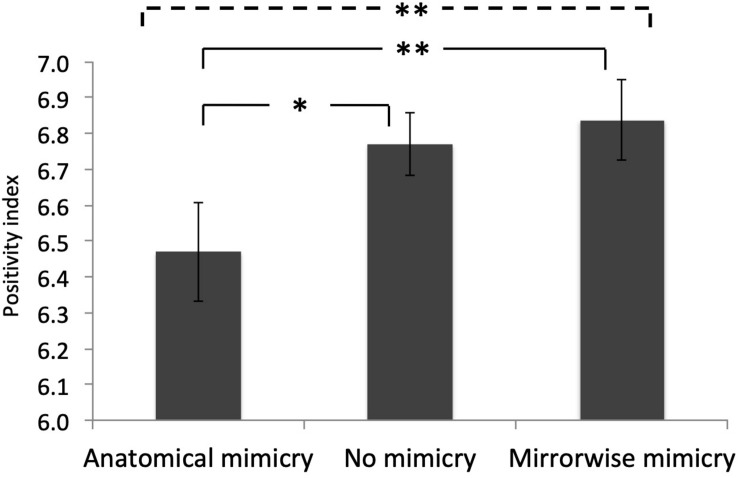
Average Positivity Index scores for each mimicry condition. Error bars show SEM. The *y*-axis values are excerpted from the full range of possible values (1–9). Horizontal lines and asterisks show the statistical significance of the main effect of mimicry (dotted line) and of the significant pairwise comparisons (solid lines; * *p* < 0.05, ** *p* < 0.01).

Differences across mimicry conditions were first analyzed using an omnibus linear regression, with subjects modeled as a repeated random effect, which showed that Mimicry Type (Anatomical Mimicry, No Mimicry, Mirror Mimicry) was a significant predictor of participants’ Positivity Index scores (Wald *χ*^2^(2) = 8.99, *p* = 0.01; Figure 2). Mirrorwise mimicry led to the highest Positivity Index scores, and Anatomical mimicry to the lowest scores.

Next we conducted a more conservative analysis, controlling for gender by adding the main effect of Gender and the interaction of Gender by Mimicry Type to the regression model (Chartrand and Bargh, 1999). Due to random assignment, the proportions of men and women were not identical across mimicry conditions (see Supplementary Table 1). Since VIRTUO was male, and men and women may differ in their social alignment behavior (Pardo, 2006) a gender imbalance across conditions could in principle affect results. Importantly, however, the main effect of Mimicry Type remained highly significant when the effect of Gender and the interaction of Gender and Mimicry Type were controlled (Wald *χ*^2^(2) = 11.21, *p* = 0.004). There was also a main effect of Gender, indicating that Positivity Index scores by women were higher, overall (Men: Mean = 6.04 ± 0.17; Women: Mean = 6.61 ± 0.11; Wald *χ*^2^(1) = 9.70, *p* = 0.002), but the effect of Gender did not interact with the effect of Mimicry Type (Wald *χ*^2^(2) = 2.92, *p* = 0.23).

We then conducted pairwise comparisons between pairs of mimicry conditions, controlling for Gender (which produced a significant main effect in the full model) but dropping the interaction of Gender by Mimicry Type (which was not significant in the full model). Of primary interest, positivity ratings in the Anatomical Mimicry condition were significantly *lower* than in the No Mimicry baseline condition: Anatomical mimicry made participants feel *more negatively* about VIRTUO (Wald *χ*^2^(1) = 4.18, *p* = 0.04; Figure 1, left columns). Likewise, Mirrorwise Mimicry led to significantly higher positivity scores than Anatomical Mimicry (Wald *χ*^2^(1) = 7.96, *p* = 0.005; Figure 2, outer columns), establishing for the first time that perspective determines the social consequences of mimicry.

The final pairwise comparison, between the Mirrorwise Mimicry and No Mimicry conditions, was not of interest with respect to the main goals of our study, which were to evaluate the social effects of Anatomical Mimicry (a) relative to Mirrorwise Mimicry (Figure 2, outer columns), a direct test of the effect of mimicry perspective, and (b) relative to a No Mimicry Baseline condition (Figure 2, left columns), a test of the social effect of anatomical mimicry, per se. The difference between Mirrorwise Mimicry and our No Mimicry baseline (i.e., the classic positive social effect of mimicry) trended in the expected direction, but was not significant according to our planned analysis (Wald *χ*^2^(1) = 1.62, *p* = 0.20; Figure 2, right columns).

Finding only a trend in the predicted direction for this pairwise comparison does not compromise our results of interest, but it bears discussion given that the positive effects of mirrorwise mimicry have been reported repeatedly, and have even been shown previously with head movement mimicry in VR (Bailenson and Yee, 2005, n.b., these results are not directly comparable to ours because a different dependent measure was used). Notably, unlike most previous studies testing for social effects of mimicry, our study did not rely on a “baseline” condition to measure the effect of primary interest: In principle, our study could have omitted the No Mimicry condition, which adds further information about our effect of interest but is not necessary for testing whether Anatomical and Mirrorwise mimicry have different social effects. This feature of the experiment is a considerable strength insomuch as our Anatomical and Mirrorwise mimicry conditions were matched in every way except for the feature of interest: spatial perspective. By contrast, it is nearly impossible to exactly match the Mimicry and No-Mimicry conditions (in our study and others), raising questions about the extent to which the various no-mimicry conditions that have been used served as appropriate baselines, and how these “baseline” conditions may have affected previously reported results.

The “baseline” condition that has been used most commonly is potentially problematic, in at least three ways. Experimenters often ask confederates to mimic particular aspects of participants’ behavior in the mimicry condition, and to not mimic them in the “baseline” condition (Chartrand and Bargh, 1999). Yet, for multiple reasons, this common procedure is likely to exaggerate the reported positive effects of mimicry. First, it is not possible to instruct confederates to perform the mimicry or no-mimicry manipulations while keeping them blind to the difference between conditions; awareness of the mimicry manipulation could lead to experimenter expectancy effects. Second, there may be an inherent asymmetry in the naturalness of mimicking vs. not mimicking a participant: Mimicking requires consciously engaging in a common, natural behavior, whereas not mimicking requires consciously *inhibiting* a common, natural behavior. Inhibiting a natural behavior may be more difficult than performing it, and this interactional difficulty for the confederate may be noticeable by the participants. Third, experimenters intend the mimicry condition to be a “treatment” condition, and the no-mimicry condition to be a “no-treatment” control condition. Yet, assuming that unconscious mimicry is ubiquitous in normal behavior (indeed, this is the central claim of the Chameleon Effect literature), exposing participants to a no-mimicry social interaction is not, in fact, the absence of a treatment: Interacting with someone who refuses to mimic is, itself, a behavioral treatment. Therefore, the Mimicry and No Mimicry conditions that are used most commonly should not be considered to be a “treatment” and a “control”; rather, they should be considered to be *two contrasting treatments*, which might be appropriately called the Plus-Mimicry and Minus-Mimicry conditions. These two behavioral interventions should be predicted to have opposite social effects: Relative to a hypothetical “natural baseline-level” of spontaneous mimicry, the Plus-Mimicry condition should have positive effects, and the Minus-Mimicry condition should have negative effects. If so, the standard mimicry/no-mimicry paradigm includes a clear source of Type I error, which could generate positive results of mimicry spuriously, or amplify the reported effect sizes. At a minimum, it is unclear to what extent previous results show a social benefit of the Plus-Mimicry condition or a social cost of the Minus-Mimicry condition.

Our task reduced or eliminated all of these concerns. First, VIRTUO was impervious to experimenter expectancy effects. Second, there was no difference in the “difficulty” with which VIRTUO could render participants’ head movements across our three mimicry conditions. Third, our No Mimicry condition, in which VIRTUO rendered a previous participant’s spontaneous movements, did not rely on a confederate stifling their natural mimicry behavior. As such, it is possible that our Mirrorwise Mimicry vs. No Mimicry effect was weaker than expected because our study reduced or eliminated sources of Type I error that were present in previous studies. Most importantly, as noted above, the main conclusion of the present study concerning the effect of mimicry perspective (Mirrorwise vs. Anatomical) does not rely on a comparison between our mimicry conditions and any “baseline” condition.

**FIGURE 3:**
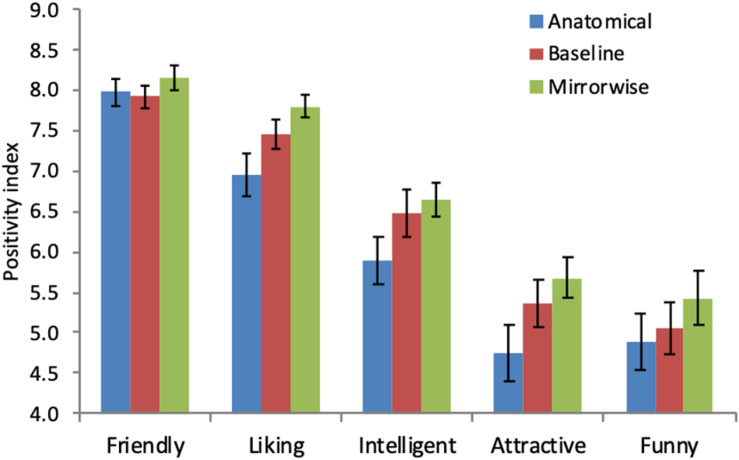
Average Positivity Index scores for each measure in each mimicry condition. Error bars show SEM. The *y*-axis values are excerpted from the full range of possible values (1–9).

Setting aside these in-principle considerations, the details of our Mirrorwise Mimicry vs. No Mimicry comparison offer some reassurance that our results (although not statistically significant in our planned analysis) showed essentially the same pattern observed in many previous studies (Chartrand and van Baaren, 2009). First, as shown in Figure 3, positivity ratings were numerically higher in the Mirrorwise condition than in the baseline condition for each of the five questions that composed our Positivity Index, individually.

Second, a small (and justifiable) change to our regression model yields a result that is more clearly in line with expectations. As reported above, we conducted pairwise analyses controlling for the main effect of Gender, since this main effect was significant in the full three-condition model. We dropped the interaction of Gender with Mimicry Condition from the pairwise comparisons, since this interaction was not significant in the full model. Yet, it is still possible that Gender could interact with Mimicry Condition to affect Positivity ratings for certain pairs of conditions. To explore this possibility *post hoc*, we tested for the difference between Mirrorwise Mimicry and No Mimicry controlling for both the main effect of Gender and the interaction of Gender by Mimicry condition: According to this analysis, the difference between Mirrorwise Mimicry and No Mimicry was on the cusp of statistical significance (Wald *χ*^3^ (1) = 3.56, *p* = 0.056).

Finally, positivity ratings were significantly higher after Mirrorwise Mimicry than after No Mimicry according to a *post hoc* nonparametric test. Upon inspecting the data in Figure 3 we noted that ratings for one of the five positivity measures, *Friendly*, were near ceiling: This was a possible cause of the unexpected lack of a significant difference between the Mirrorwise and No Mimicry conditions since a ceiling effect may have obscured the *magnitude* of the difference between these mimicry conditions, reducing its statistical significance in our planned parametric test. Accordingly, we conducted *post hoc* nonparametric sign tests (which are sensitive to the direction of the differences between conditions, but not their magnitudes), which showed that positivity ratings in the Mirrorwise Mimicry condition were significantly higher than in both the Anatomical Mimicry and the No Mimicry conditions (*z* = 2.24, *p* = 0.025, two-tailed, for both comparisons). In light of the consistent qualitative pattern and the results of the *post hoc* analyses, we hesitate to interpret these data as *failing* to show the expected positive effect of mirrorwise mimicry. Finally, returning to the main goals of this study, the effect of mimicry perspective is not indexed by the difference between Mirrorwise Mimicry and baseline, but rather by the difference between Mirrorwise Mimicry and Anatomical Mimicry: This difference was highly significant.

## General Discussion

Bodily mimicry can influence people’s attitudes about their conversational partners – even virtual partners. Here we show that the spatial perspective adopted by a mimicker can determine whether mimicry has a positive or a negative effect on the mimickee. Participants who were mimicked anatomically rated VIRTUO significantly more *negatively* than participants who were mimicked mirrorwise, or not mimicked at all. In face-to-face conversation, mirrorwise and anatomical mimicry had opposite social consequences. These results suggest that motor mimicry’s positive consequences depend on spatial similarity between the mimicker and mimickee, not similarity in motor circuits or effectors, which is greater for anatomical mimicry than for mirrorwise mimicry.

This study is the first to demonstrate that anatomical mimicry can cause the mimickee to evaluate the mimicker more negatively: a *Reverse-Chameleon Effect*. These data are broadly consistent with those of an observational study testing relationships between students’ rapport with their professors and the rate at which they adopted body postures similar to their professors’. LaFrance and Broadbent (1976) found a positive correlation between rapport and the rate of mirrorwise mimicry, but no significant association between rapport and anatomical mimicry (Bavelas et al., 1988). More broadly, these data are consistent with studies showing that the effect of mimicry is not always positive, and can depend on the specifics of the social situation (Stel et al., 2010; Liu et al., 2011) and the personality of the mimicker (Leander et al., 2012) or the mimickee (Kavanagh et al., 2011; Stel et al., 2011; Sparenberg et al., 2012).

Why might mirrorwise mimicry have positive consequences and anatomical mimicry negative consequences in the mind of the mimickee? We consider four possible explanations based on (1) contingency of behaviors, (2) perceptual oneness, (3) perceptual fluency, and (4) signaling cooperation.

On the first possibility, mimicry could have positive social consequences because it creates a *contingency between interlocutors’ behaviors*, making the behavior the mimicker more predictable to the mimickee (Catmur and Heyes, 2013). The present results do not support this proposal: VIRTUOs movements in the mirrorwise and anatomical mimicry conditions shared the same contingency relationships with the subjects’ movements. If contingency were driving the social effects of mimicry then subjects’ ratings of VIRTUO should have been equally positive across our mimicry conditions; contingency cannot explain why mirrorwise mimicry had positive effects but anatomical mimicry had negative effects. Thus, the present results militate against contingency as a driver of mimicry’s positive (or negative) social effects.

Second, Bavelas et al. (1988) proposed that the mimicker’s actions are designed to communicate a message to the mimickee: “I am with you” or “I am like you” (Chartrand and Bargh, 1999). Bavelas and colleagues suggested that mirrorwise mimicry can convey this message, but anatomical mimicry cannot, because only mirrored actions can be perceived in terms of the Gestalt principles of similarity and common fate: indices of *perceptual “oneness”* between mimicker and mimickee, and therefore of social unity. Although it is possible that mirrorwise mimicry communicates the message “I am like you” to mimickees as Bavelas et al. (1988) suggest, we note that it is unlikely that this message is communicated via principles of gestalt visual perception that give rise to a sense of perceptual oneness, for a simple reason: In many situations, including the present experiment, mimickees are unable to *see* their own actions that are being mimicked. In such situations, the effects of mimicry must depend on a match (or mismatch) between the mimickee’s visual perception of the mimicker’s actions and their *proprioceptive sense* of their own body’s position – not on Gestalt visual principles.

On a third possibility, from early childhood, people have a tendency to spontaneously mimic others mirrorwise (Wapner and Cirillo, 1968; Bavelas et al., 1988). Mimickees should be most accustomed to seeing mimickers adopting this perspective, making mirrorwise mimicry more predictable and easier to process visually than anatomical mimicry. If so, we suggest that differences in *processing fluency* could account for the opposite effects of the two imitation perspectives, since in many circumstances, fluency leads to positive evaluations and disfluency to negative evaluations (Casasanto and Chrysikou, 2011; Oppenheimer, 2008; Reber et al., 2004).

Finally, face-to-face mirroring increases overlap in the trajectories of the actions that mimickers and mimickees perform. To clarify, anatomical mimicry increases overlap in the effectors used to perform actions (since both parties are using the same body parts, as opposed to using contralateral homologs), but decreases overlap in the spatial trajectories and locations of those actions compared to mirrorwise mimicry. Outside of the laboratory, conversations are often situated in an environment populated with manipulable objects, and take place because one or both parties want to achieve some goal. This goal may entail interacting with the objects at hand. Suppose that one person wants to help the other person act upon an object located to one person’s right. If they start out facing each other, when they reach for the object they must move in mirrorwise fashion.

We suggest that the positive social consequences of mirrorwise mimicry – and the negative consequences of anatomical mimicry – could arise from the fact that mimicking mirrorwise increases the mimicker’s readiness for cooperative joint actions (Clark, 1996; Sebanz et al., 2006) whereas anatomical mimicry decreases readiness to cooperate. Many of the actions that people mimic, including the head movements we manipulated in this study, may not be not goal directed, but habitually mimicking prepares the mimicker to cooperate with those actions that are goal directed. According to this proposal, which we call the *cooperation hypothesis*, mimicry that increases the mimicker’s readiness to perform cooperative actions could have a positive influence on the mimickee whether or not mimicry enhances visual blending-in (Chartrand and Bargh, 1999) or perceived oneness (Bavelas et al., 1988).

The present data are inconsistent with the first two possible explanations for the perspective-dependence of mimicry’s effect (contingency of behaviors, gestalt visual perception of oneness), but these results are consistent with the latter two possibilities that we propose (perceptual fluency, signaling readiness to cooperate). The fluency and cooperation hypotheses are mutually compatible, but in principle they can be evaluated individually. Testing these candidate explanations remains a project for ongoing research.

## Conclusion

Whereas mirrorwise bodily mimicry generally has positive social consequences, we show that anatomical mimicry can have negative consequences. These results suggest that visuo-spatial similarity between the mimicker and mimickee underlies mimicry’s positive effects: not similarity in terms of motor plans and effectors, which is greatest for anatomical mimicry during face-to-face conversations. People who use mimicry strategically to win customers (Tanner et al., 2008) or build affiliation with patients in psychotherapy (Scheflen, 1964) should consider that the spatial perspective they adopt may determine whether mimicry has a positive or negative effect.

## Data Availability Statement

The datasets generated for this study are available as Supplementary Material (Supplementary Data Sheet 2).

## Ethics Statement

The studies involving human participants were reviewed and approved by the Max Planck Institute for Psycholinguistics, Nijmegen, NL, United States. The patients/participants provided their written informed consent to participate in this study.

## Author Contributions

DC and LC designed the study. TG collected the data. DC performed the statistical analyses and wrote the first draft of the manuscript. All authors contributed to the manuscript revision, and read and approved the submitted version.

## Conflict of Interest

The authors declare that the research was conducted in the absence of any commercial or financial relationships that could be construed as a potential conflict of interest.

## Footnotes

1 www.adobe.com

2 www.ar-tracking.com

3 www.worldviz.com
