# Supplementary material for: The Reverse Chameleon Effect: Negative Social Consequences of Anatomical Mimicry
Source: Front Psychol. 2020 Jul 31;11:1876. doi: 10.3389/fpsyg.2020.01876 (PMC7411309; doi:10.3389/fpsyg.2020.01876)
Supplement: Supplementary file 1 [file Data_Sheet_1.PDF]

Supplementary materials for Casasanto, Staum Casasanto, Gijssels, & Hagoort (2019), *The Reverse Chameleon Effect: Negative social consequences of anatomical mimicry.*

VIRTUO's script (English)
Hi, I'm VIRTUO! I'm trying to learn to be more like a human.
Right now I'm learning about things you buy at the grocery store.
I've never been to a grocery store, but I've been reading about what it's like.
I've learned a little bit about the kinds of things you can find there.
I still don't understand everything, though. Would you take a ride with me and answer some of my questions?

This item doesn't seem to match any of my files.
Can you tell me what this is? Can you tell me about the climate you need to grow bananas? How do you tell when a banana is ready to eat? They say fruit is good for you. What are the health benefits of eating bananas? I know you're supposed to wash apples before you eat them. How come you don't have to wash a banana before you eat it?

My data files suggest this is ketchup, is that right? I'm not programmed with taste buds, but I'd like to try to understand what things taste like. Could you tell me what ketchup tastes like?
What do you think are some of the ingredients in ketchup? I bet ketchup can be messy. If you get ketchup on your clothes, how do you get it out? I've heard you can eat ketchup on hot dogs. What is a hot dog like?

Is this a lightbulb? I have a joke for you. How many artificially intelligent virtual agents does it take to screw in a lightbulb? None! The virtual world is already adequately lit! In the olden days, people used candles for light. Why are light bulbs better than candles? What is the metal part at the bottom for? I hear you need light when you're reading. When else do you need a light bulb?

I think this is toothpaste, is that right? I hear some tooth paste is whitening. Why do people want to do use that? What happens if you don't brush your teeth? What's the difference between brushing and flossing? I understand it's important not to waste any of the things that you buy. What's the best way to get all the toothpaste out of the tube?

This says it's cat food, is that right? I understand that cats have digestive systems much like humans. Why can't a cat eat people food? What other animals need a special food to eat? What did cats eat before people started taking care of them? What is the difference between cat food and dog food?

I recognize this -- this is beer, isn't it? Someday maybe my programming will include drinking. After you drink a beer, what do you do with the bottle? What is the difference between beer and wine? I know there are health reasons why some people can't drink beer. Why shouldn't pregnant women drink it?
****
OK, let's see what's ahead. Thanks. Let's see what's next. I see, let's see what's next. OK, let's drive a little farther. That's good to know. Let's go to the next item. That's all the training we have scheduled for today. Thanks -- I've learned a lot.

The experimenter is going to return you to the human world now, but I hope you'll come back and visit me another time. I'm sorry, I don't understand what you just said -- I'm still learning. Let's move on.
****

VIRTUO's script (Dutch)
Hallo, ik ben VIRTUO! Ik probeer steeds meer op een mens te lijken.
Momenteel ben ik aan het leren over wat je bij de kruidenier kunt kopen.
Ik ben nog nooit in een kruidenierswinkel geweest, maar ik heb er wel over gelezen.

Ik heb wel wat geleerd over wat je daar allemaal kunt kopen.
Maar ik begrijp nog lang niet alles.
Wil je een stukje met me gaan rijden om een paar vragen te beantwoorden?

Dit item klopt met geen van mijn files.
Kun je me zeggen wat het is?
Kun je me iets zeggen over het klimaat waarin bananen groeien?
Hoe weet je wanneer een banaan rijp is om te eten?
Ze zeggen dat fruit goed voor je is. Waarom zijn bananen gezond?
Ik weet dat je appels moet wassen voordat je ze eet. Waarom hoef je een banaan niet te wassen voordat je hem eet?

Mijn gegevens doen denken dat dit ketchup is. Klopt dat?
Ik ben niet geprogrammeerd met smaakpupillen, maar ik zou toch graag weten hoe dingen smaken.
Kun je me vertellen hoe ketchup smaakt?
Wat zijn volgens jou de ingrediënten van ketchup?
Ik denk dat ketchup een hoop rotzooi kan maken. Als je ketchup op je kleren krijgt, hoe krijg je het er weer uit?
Ik heb gehoord dat je ketchup op een hot dog kan eten. Wat is een hot dog?

Is dit een gloeilamp?
Ik heb een grap voor je. Hoe veel kunstmatig intelligente virtuele personen heb je nodig om een gloeilamp in te draaien?
Geen één! De virtuele wereld is al voldoende verlicht.
Vroeger gebruikten de mensen kaarsen als verlichting. Waarom zijn gloeilampen beter dan kaarsen?
Waarvoor dient het metalen deel aan de onderkant?
Ik hoor dat je licht nodig hebt om te lezen. Waar heb je verder nog gloeilampen voor nodig?

Volgens mij is dit tandpasta. Klopt dat?
Ik heb gehoord dat sommige tandpasta's je tanden wit maken. Waarom willen mensen dat?
Wat gebeurt er wanneer je je tanden niet poetst?
Wat is het verschil tussen poetsen en flossen?
Ik heb begrepen dat het belangrijk is de dingen die je koopt niet te verspillen. Wat is de beste manier om alle tandpasta uit een tube te krijgen?

Hierop staat dat het kattenvoer is. Klopt dat?
Ik heb begrepen dat de manier waarop katten hun voedsel verteren veel lijkt op die waarop mensen dat doen. Waarom kan een kat geen mensenvoer eten?
Welke dieren hebben verder nog speciaal voer nodig om te eten?
Wat aten katten voordat de mensen begonnen voor ze te zorgen?
Wat is het verschil tussen kattenvoer en hondenvoer?

Dit kan ik herkennen. Het is bier, toch?
Misschien zal mijn programma het me eens mogelijk maken om te drinken. Als je bier gedronken hebt, wat doe je dan met de fles?
Wat is het verschil tussen bier en wijn?
Ik weet dat sommige mensen om gezondheidsredenen geen bier mogen drinken. Waarom mogen zwangere vrouwen geen bier drinken?

****
OK, laten we eens kijken wat er nog te doen staat.
Dank je wel. Laten we eens kijken wat er nu volgt.
Juist. Wat volgt er nu?
OK, laten we even verder gaan.
Goed om te weten. Laten we nu naar het volgende item gaan.

Dit is dan de hele training die voor vandaag gepland staat.
Dank je wel. Ik heb een hoop geleerd.
De testleider gaat je nu naar de mensenwereld terugbrengen, maar ik hoop dat je nog eens terugkomt om me op te zoeken.
Sorry, dat heb ik niet begrepen. Ik ben nog aan het leren, snap je? Laten we verder gaan.
******
