# Supplementary material for: The Reverse Chameleon Effect: Negative Social Consequences of Anatomical Mimicry
Source: Front Psychol. 2020 Jul 31;11:1876. doi: 10.3389/fpsyg.2020.01876 (PMC7411309; doi:10.3389/fpsyg.2020.01876)
Supplement: Supplementary file 3 [file Data_Sheet_3.PDF]

Supplementary table 1 for:

Casasanto, D., Staum Casasanto, L., Gijssels, T. & Hagoort, P. (2020). The reverse chameleon effect: Negative social consequences of motor mimicry. *Frontiers in Psychology.*

Supplementary table 1. Means and standard errors (in parentheses) for each condition by gender.

	Anatomical (n = 11)	Baseline (n = 15)	Mirrorwise (n = 11)
Men	6.10 (.31)	6.49 (.13)	6.64 (.24)
	Anatomical (n = 28)	Baseline (n = 23)	Mirrorwise (n = 29)
Women	6.62 (.15)	6.95 (.10)	6.91 (.13)
